# First-Day-of-Life Rectal Swabs Fail To Represent Meconial Microbiota Composition and Underestimate the Presence of Antibiotic Resistance Genes

**DOI:** 10.1128/spectrum.05254-22

**Published:** 2023-04-25

**Authors:** S. Graspeuntner, M. Lupatsii, L. Dashdorj, A. Rody, J. Rupp, V. Bossung, C. Härtel

**Affiliations:** a Department of Infectious Diseases and Microbiology, University of Lübeck, Lübeck, Germany; b German Center for Infection Research (DZIF), Partner Site Hamburg-Lübeck-Borstel-Riems, Lübeck, Germany; c Department of Obstetrics and Gynecology, University Hospital of Schleswig-Holstein, Lübeck, Germany; d Department of Obstetrics, University Hospital of Zurich, Zurich, Switzerland; e Department of Pediatrics, University Hospital of Würzburg, Würzburg, Germany; University of Guelph College of Biological Science

**Keywords:** microbiome, rectal swabs, meconium, resistance genes, gut microbiota, human microbiota, newborns, antibiotic resistance

## Abstract

The human gut microbiome plays a vital role in health and disease. In particular, the first days of life provide a unique window of opportunity for development and establishment of microbial community. Currently, stool samples are known to be the most widely used sampling approach for studying the gut microbiome. However, complicated sample acquisition at certain time points, challenges in transportation, and patient discomfort underline the need for development of alternative sampling approaches. One of the alternatives is rectal swabs, shown to be a reliable proxy for gut microbiome analysis when obtained from adults. Here, we compare the usability of rectal swabs and meconium paired samples collected from infants on the first days of life. Our results indicate that the two sampling approaches display significantly distinct patterns in microbial composition and alpha and beta diversity as well as detection of resistance genes. Moreover, the dissimilarity between the two collection methods was greater than the interindividual variation. Therefore, we conclude that rectal swabs are not a reliable proxy compared to stool samples for gut microbiome analysis when collected on the first days of a newborn’s life.

**IMPORTANCE** Currently, there are numerous suggestions on how to ease the notoriously complex and error-prone methodological setups to study the gut microbiota of newborns during the first days of life. Especially, meconium samples are regularly failing to yield meaningful data output and therefore have been suggested to be replaced by rectal swabs as done in adults as well. We find this development toward a simplified method to be producing dramatically erroneous results, skewing data interpretation away from the real aspects to be considered for neonatal health during the first days of life. We have put together our knowledge on this critical aspect with careful consideration and identified the failure of rectal swabs to be a replacement for sampling of meconium in term-born newborns.

## INTRODUCTION

Rising interest in microbiome studies uncovers the crucial importance of the gut microbiota in health and disease (1). After birth, the first days of a newborn’s life provide a unique window of opportunity for gut microbiome establishment, which impacts long-term health and development of the infant (2, 3). Thus, we and others have shown that multiple factors, for instance, antibiotic administration prenatally (4, 5), intrapartum (6–9), and postnatally (10), mode of birth (11), type of feeding (12, 13), gestational age (14), and maternal vertical transmission (15), strongly impact the formation and stability of the newborn’s microbiota. To advance current knowledge about factors influencing early gut colonization and microbiome development in infants, standardized and easy sampling techniques are needed to increase comparability of the studies and participant recruitment rates. Currently, collecting stool samples is known to be the “gold standard” in gut microbiome studies (16–18). Although stool sampling enables us to obtain a large amount of microbial biomass and is suitable for self-sampling by study participants in a nonclinical setting, several disadvantages emphasize the need for developing alternative sample collection techniques (18). Stool sampling is hardly standardized and hampered by a high contamination risk, and the definition of specific sampling time points as well as sampling in patients with severe health conditions (19) may be complicated. Furthermore, challenges in transport and storage (20) as well as the participant’s discomfort level (21) are known drawbacks of stool sampling. One of the alternatives is the use of rectal swabs, which enables sampling at exact time points, eases collection, storage, and preservation of the samples, increases standardization, and limits contamination risk (22). Several studies indicated collection of rectal swabs as a reliable proxy for stool samples in adults (18–20, 22, 23). In comparison to sample collection from adult stool, sampling from infants possesses even more challenges as defecation frequency varies greatly among newborns (24), complicating sampling at certain time points. Furthermore, constipation’s prevalence is higher in the first years of life (25). To our best knowledge, currently only a few studies have compared the use of rectal swabs and stool samples in gut microbiome analysis in infants (26, 27). Reyman and colleagues screened 21 sample pairs on the first 2 days of life and 110 sample pairs in the first week after birth in a group of term-born infants with suspected early-onset sepsis (26). The study reported high comparability between the two collection methods; however, the number of observed species was significantly higher for meconium samples at the age of 6 days. Another study assessed the feasibility of rectal swab use in children at an age ranging from 3 months to 4.4 years, observing similarity between rectal swabs and stool samples (27).

An important point which was not addressed in previous studies is the detection of resistance genes in the gut microbiome of newborns. Deepening analysis of the gut resistome development is essential for understanding the emergence of antimicrobial resistance and preservation of microbial communities in the gut. In this study, we compare rectal swab and meconium sample collection methods in a cohort of term Caesarean section-born infants aged 1 to 3 days to evaluate whether rectal swabs could be used as a reliable proxy for studying the gut microbiome. We further compare corresponding presences of selected resistance genes in the microbiome of newborns.

## RESULTS

In a previous study, we evaluated the impact of antimicrobial prophylaxis timing on the development of the infant’s gut (9). From the participants of this study, we were able to obtain 13 rectal swabs taken on the 1st day of life which had matching meconium samples available. This enabled us to perform a comparative analysis of the sampling strategies at the beginning of life.

### Alpha and beta diversity display significantly different patterns for two sampling types.

**FIG 1:**
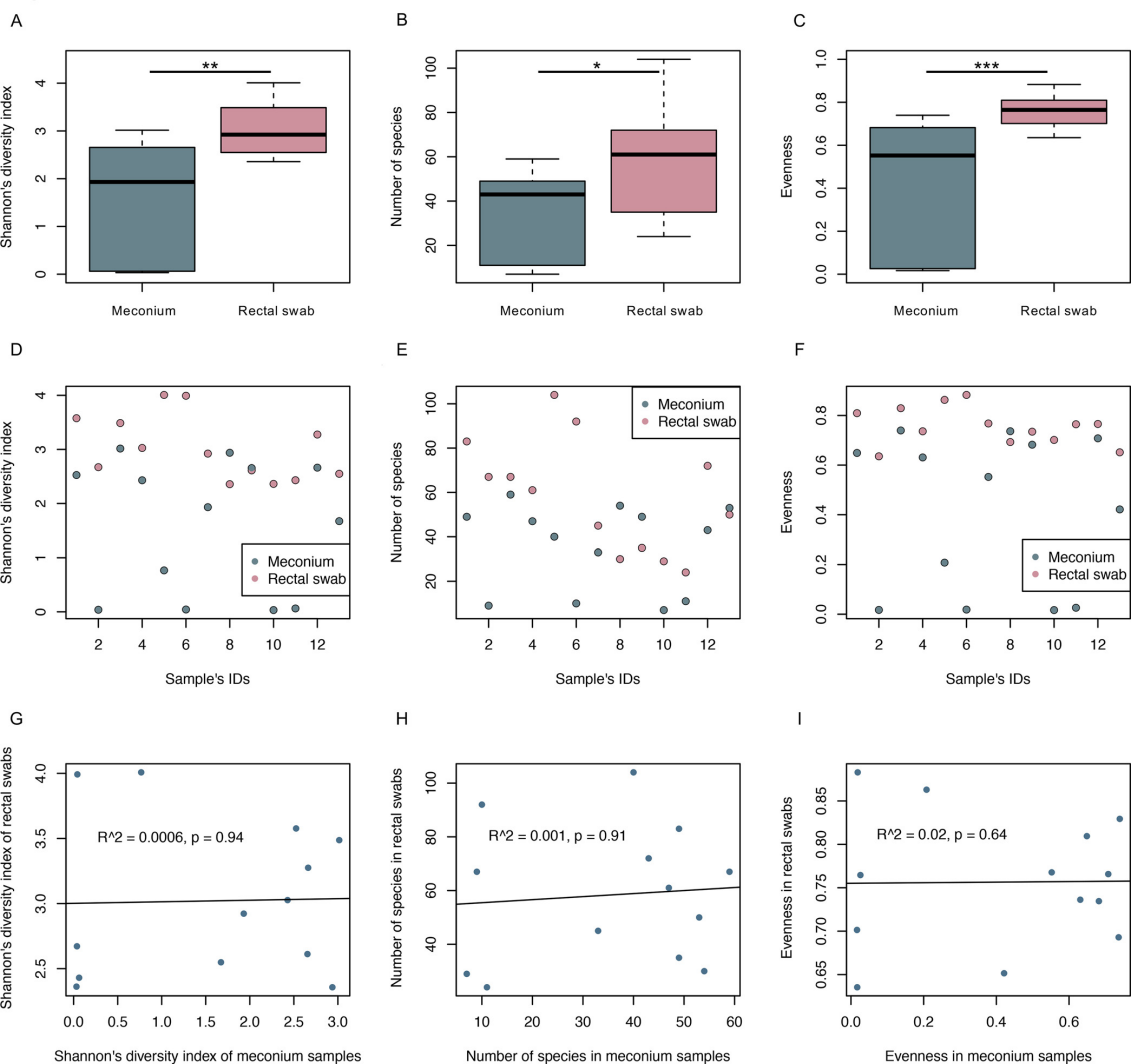
Alpha diversity of the newborn’s gut microbiome differs greatly between the two sample collection approaches. Comparison of alpha diversity measurements assessed by calculating Shannon’s diversity index (A), detected number of species (B), and evenness index (C) for meconium samples and rectal swabs. Degree of significance was evaluated using the Wilcoxon rank sum test (*, *P* < 0.05; **, *P* < 0.01; ***, *P* < 0.001). (D to I) Depiction of alpha diversity measures for both sampling types on a sample-by-sample basis (D to F) as well as correlation between alpha diversity measurements for the two collection approaches (G to I) indicated lack of similarity between the sampling approaches.

Assessment of the alpha diversity measurements indicated significant differences between rectal swabs and meconium samples, Shannon’s diversity index (*P* < 0.01), detected number of species (*P* < 0.05), and evenness index (*P* < 0.001) were higher for rectal swab samples (Fig. 1A to C). Pairwise plotting of alpha diversity measures of both sampling methods for each of the participants depicts differences (Fig. 1D to F) which revealed no correlation between stool samples and rectal swabs (Fig. 1G to I; Pearson’s correlation index, *R*^2^ = 0.0006, *P* = 0.94; *R*^2^ = 0.001, *P* = 0.91; *R*^2^ = 0.02, *P* = 0.64, respectively).

**FIG 2:**
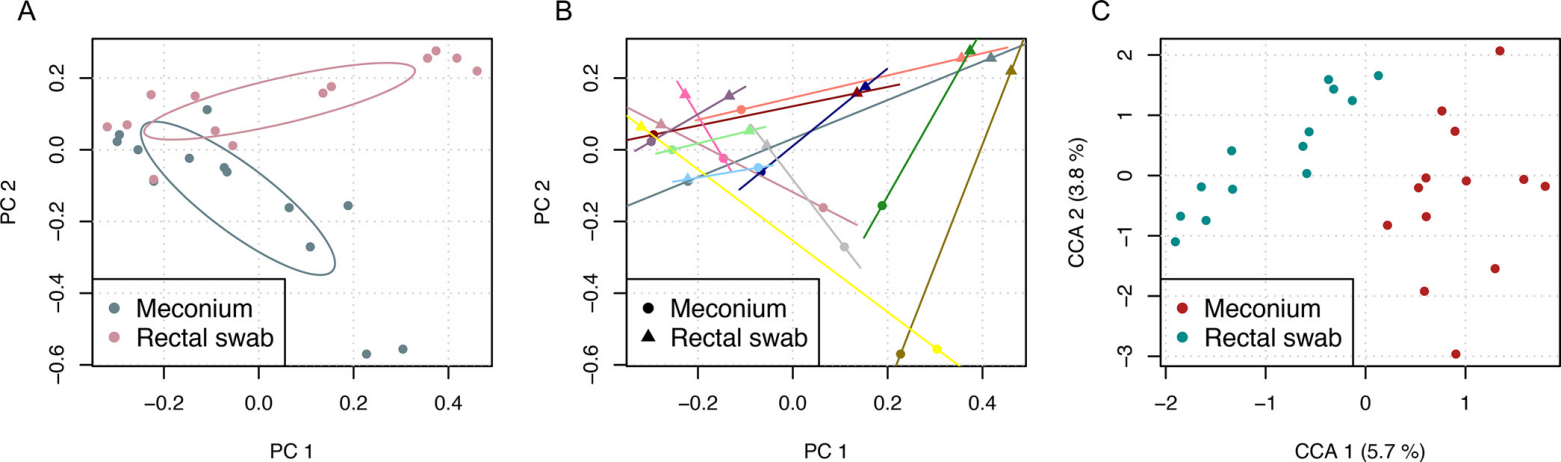
Beta diversity analysis reveals distinct clustering for both sample collection methods. (A and B) Beta diversity was compared by computing principal-coordinate analysis (A) depicting distinct clusters for two sample groups (permutational multivariate analysis of variance using distance matrices, *P* < 0.01) and with assignment to variable color coding for the matched sample pairs (B). (C) Constrained correspondence analysis (CCA) proved the significant contribution of the sampling method (*P* < 0.01) and the nonsignificant impact of interindividual variation (*P* > 0.05).

Comparison of beta diversity via computation of principal-coordinate analysis depicted distinct clustering for both sample types with only slight overlap (Fig. 2A). Calculation of permutational multivariate analysis of variance using distance matrix test revealed significant difference in beta diversity composition between the two groups (*P* < 0.01). To compare the impact of sampling type with the interindividual variation, pairing of the samples was depicted (Fig. 2B). The results show that meconium samples and rectal swabs from the same infant do not cluster. To further characterize the effect of interindividual variation, constrained correspondence analysis was performed with constraining the data set for both sample type and participant identifier (ID). Distinct clustering along the *x* axis assigned to sample type can be observed (Fig. 2C), and type of sampling accounted for 5.7% of constrained variation in the data set whereas interindividual variation explained 3.8% of variation. Analyses of variance like the permutation test displayed significance (*P* < 0.01, with 999 permutations) for impact of sampling type but not for the interindividual variance (*P* > 0.05, with 999 permutations).

### Paired meconium samples and rectal swabs are distinct in their taxonomic compositions.

**FIG 3:**
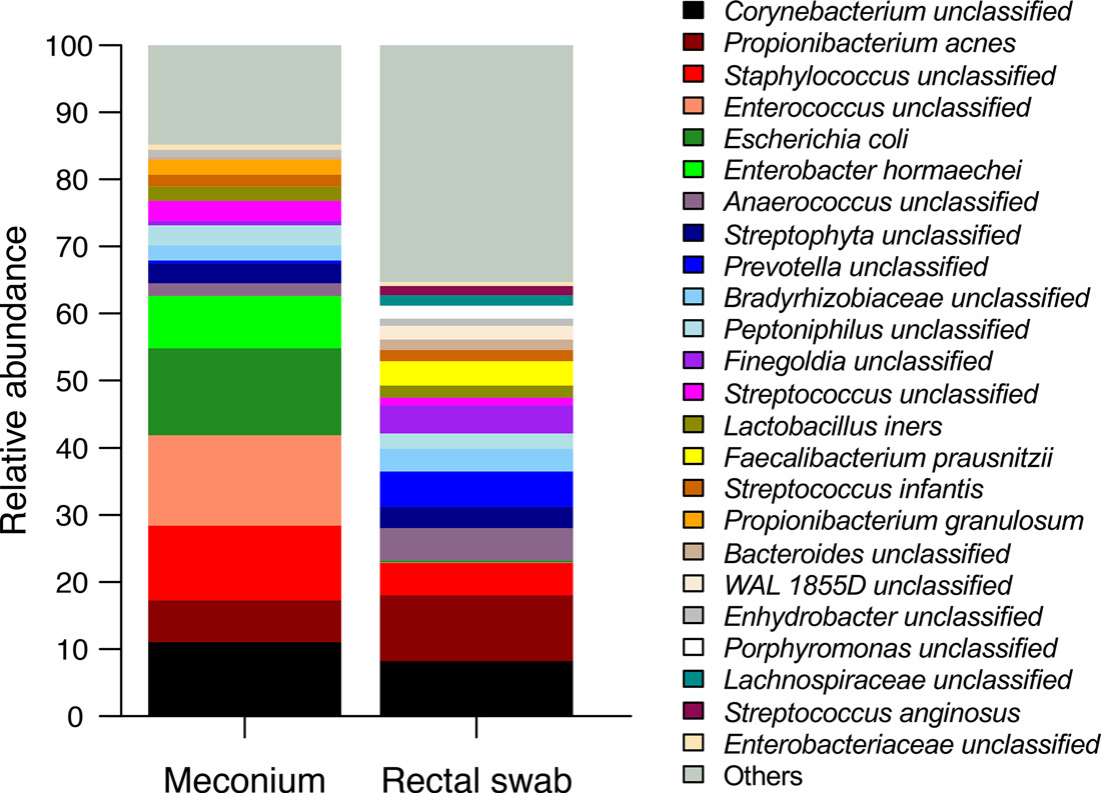
Comparison of the taxonomic composition in meconium samples and rectal swabs indicates different microbial composition between the sampling strategies. Relative abundance of the 24 most prevalent taxa on the genus level is given in both sampling types. Taxa with higher abundance are listed at the top of the key.

**FIG 4:**
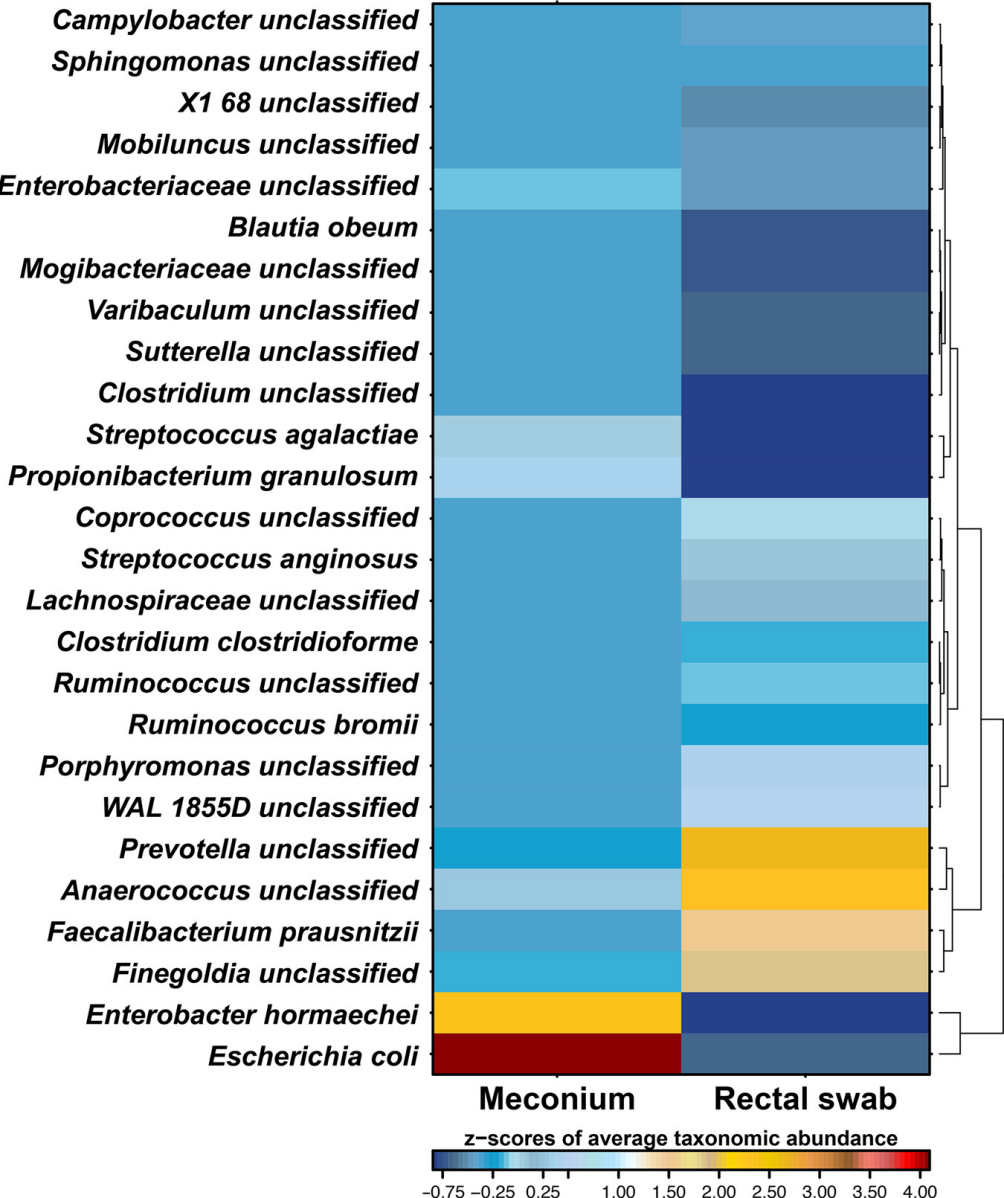
Heatmap of taxa displaying significant differences between two sampling methods. Significance was calculated via pairwise Wilcoxon rank sum test (*P* < 0.05, with use of false-discovery rate adjustment of *P* values). Color coding depicts the Z-scores of average taxonomic abundance.

Comparison of relative abundances of most prominent species for both sample types revealed distinct patterns for each of the groups (Fig. 3 and see Fig. S1 in the supplemental material). Meconium samples had higher levels of genera Staphylococcus, *Enterococcus*, Escherichia, Enterobacter, Streptococcus, and *Propionibacterium*, while rectal swabs had elevated abundance of *Anaerococcus*, *Prevotella*, *Finegoldia*, *Faecalibacterium*, WAL 1855D, *Porphyromonas*, *Lachnospiraceae*, Streptococcus anginosus, *Enterobacteriaceae*, *Dialister*, *Blautia*, *Coprococcus*, and *Ruminococcus*. Overall, 26 taxa had a significantly different abundance between the two groups (*P* < 0.05, adjusted for false-discovery rate) (Fig. 4 and Fig. S2). Although it is only 1/10 of 247 detected species, their cumulative abundance accounts for one-third of the microbiome composition (33.5% of relative abundance for rectal swabs and 27.9% for meconium samples), underlining the differences between groups.

### Abundance of antibiotic resistance genes differs between rectal swabs and meconium samples.

**FIG 5:**
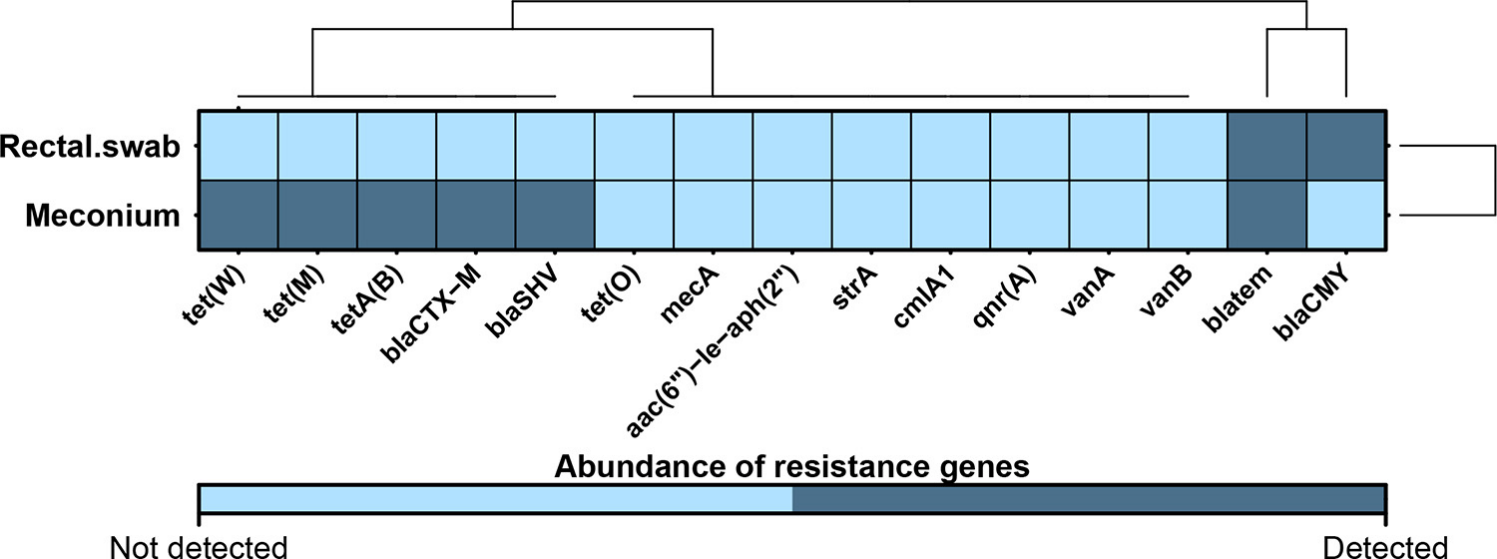
Heatmap depicting different detectabilities of antibiotic resistance genes in the sample types. Clustering was performed based on the Euclidean distance; light blue represents the absence of the resistance genes in the samples from one of the collection approaches, and dark blue represents presence in at least one of the samples of the group.

To further compare the two sampling methods, the abundance of 15 selected resistance genes was evaluated in the paired meconium samples and rectal swabs. Only two rectal swabs and two meconium samples harbored resistance genes in general. Out of 15 tested genes 7 were detected with meconium samples harboring 6 of 15 genes compared to rectal swabs harboring only 2 resistance genes. Assuming meconium samples to be resembling the true rate of resistance genes in a newborn, rectal swabs depict a positive predictive value of 0%, whereas the negative predictive value in our data set for rectal swabs was 81.82%. The two antibiotic resistance genes detected in rectal swabs were beta-lactamases *bla*_TEM_ and *bla*_CMY_, whereas in meconium samples beta-lactamases *bla*_TEM_, *bla*_CTX-M_, and *bla*_SHV_ were detected as well as genes carrying resistance to tetracyclines, *tet*(W), *tet*(M), and *tetA*(B) (Fig. 5). We were, in addition, able to isolate Enterococcus faecalis and Escherichia coli from the *bla*_SHV_-positive microbiome sample and proved the isolated E. coli to be carrying the *bla*_SHV_ gene, corresponding to the data from the microbiome sample.

## DISCUSSION

Improved knowledge on the gut microbial colonization and host-microbiome interaction early in life is a key to uncover novel therapeutic and prophylactic strategies to maintain health in vulnerable newborns. In this light, precisely validated and easy-to-apply sampling approaches are a key to comparability of and high recruitment rates for scientific studies (23). In the current analysis, rectal swabbing was compared as an alternative to stool sampling in a cohort of term Caesarean section-born infants on their first days of life. Sampling for both approaches by health care professionals in the same clinical environment as well as the possibility to obtain both sample types from the same infants under standardized sampling and storage conditions provided the opportunity for comparative analysis.

In our hands, distinct patterns in the microbiome composition as well as significant differences in alpha diversity measurements indicate high dissimilarity between rectal swabs and meconium samples. Here, we report significantly higher Shannon’s diversity index, number of species, and evenness evident for rectal swabs collected on the 1st day of life. In line with our results, one of the previously reported findings derived from sampling conducted from adult participants depicted significantly different Shannon’s diversity index values for rectal swabs and stool samples (28). Several more studies also revealed elevated levels of observed species (26) and Shannon’s diversity index (18, 23, 29) in rectal swabs. Though contrary to what we describe in our study, differences were not significant, leading some authors to the questionable conclusion that the two sampling methods yield similar outcomes. Interestingly, Turner and colleagues pointed out that alpha diversities were significantly different between stool samples and clinically collected swabs; however, if the swabs were self-obtained, the difference did not reach significance (22). In our study, all rectal swabs were collected by physicians in the hospital setting, which may increase the quality of sample acquisition as well as reducing the contamination rate, revealing a clearer difference when comparing swabs to stool samples. The current study revealed distinct patterns of the infant gut microbiome depending on the sample collection method. It is known that the Caesarean section-born infant gut microbiome tends to be dominated by skin-associated bacteria such as genera *Corynebacterium*, *Propionibacterium*, and Staphylococcus (11). These genera were also highly abundant in our data set, accounting for 28.4% of relative abundance in meconium samples and 22.9% in rectal swabs. While meconium samples harbored elevated levels of enteric bacteria (30) such as Escherichia, Enterobacter, and *Enterococcus*, rectal swabs had high relative abundances of *Finegoldia*, *Prevotella*, and *Anaerococcus*, known to be associated with infant buttock skin (31, 32). From 247 detected taxa, 26 accounting for nearly one-third of total microbial composition were found to have significantly different relative abundances in the two sampling techniques. The elevated presence of *Finegoldia*, *Porphyromonas*, *Lachnospiraceae*, and WAL 1855D (unclassified sporobacteria) in rectal swabs is in line with previous findings (18, 19, 33). In contrast to prior studies (19), the abundance of the genus *Ruminococcus* was lower in stool samples. Such observed differences between the two sampling methods have previously been explained by the fact that rectal swabs are known to be a closer representation of the mucosal microbiome whereas stool samples reflect the luminal microbial community (28, 34). However, a more crucial aspect to take into account in our case is that the timing of meconium passage in infants varies and may occur in the first 72 h after birth (35, 36), indicating that if a rectal swab is sampled prior to meconium passage, it may serve as a representation of the skin microbiome rather than of the gut microbial consortium. Taking into account the results from Reyman and colleagues (where the focus was set on close proximity of time between sample types rather than on sampling directly after birth) (26), we emphasize the importance of precise study design with appropriate sampling planning to avoid misleading results.

Comparison of beta diversities revealed higher dissimilarity between different sample types than interindividual variation if the rectal swab was sampled directly after birth. Similar observations were made by Sun and colleagues, who found a significant difference in beta diversities between matched rectal swabs and stool samples collected from 40- to 85-year-old participants with a history of colorectal polyps (28). Additionally, it was revealed that the difference between two sampling methods is present also for functional pathways of metagenomes connected to peptidoglycan, nucleic acids, sugars, and amino acid synthesis (28). Comparison of mucosal biopsy specimens with rectal swabs and stool samples in the study by Budding and colleagues confirmed distinct microbiota profiles between rectal swabs and stool samples collected from patients undergoing colonoscopy; however, differences from mucosal samples were more distinct (20). While others have published contradictory results (22), our data further suggest that the type of sampling explains more variation in beta diversity than whether the samples were obtained from the same individual. As all of the infants in the current study were born in the same hospital, the similar surroundings may have decreased interindividual variability, specifically taking into account that the microbiome of Caesarean section-born infants is highly impacted by clinical surroundings (37). In our study we had available comparative samples only for the first days of life. In this sense it is also important to note that the newborn’s microbiome is rapidly changing in the course of development (38), implying that sampling type impact may differ at different stages of life. All in all, while several studies state that rectal swabs could be a reliable substitute for stool sampling (19, 22, 26, 27, 39, 40), our results indicate that on the first days of life rectal swabbing is not a reliable proxy for early gut microbiome analysis. While this dismisses the usage of rectal swabs in newborns, rectal swabs may be considered an alternative in adults. To further pinpoint this, we currently lack close longitudinal comparative sampling to address how slowing down of changes within the microbiome over time would affect comparison of rectal swabs versus stool samples in our hands. Thus, it would also be useful to include the mother’s microbiome signatures for a full-range comparative analysis, which we cannot provide here. It further is noteworthy that stool samples are representing a mixture of microbes from different regions of the gut. Thus, in situations where mucosal microbes in particular are of relevance (e.g., mucositis), swabs may be the better alternative to stool samples independent of other considerations.

A largely neglected aspect in the quality assessment of sampling types is abundance of antibiotic resistance genes with, so far, no data available addressing differences between stool samples and rectal swabs. It was previously shown that an infant’s gut carries variable antimicrobial resistance genes (41). Moreover, an infant’s gut is more susceptible to colonization by drug-resistant bacteria as colonization resistance is not yet developed due to the microbiome not being yet established (42). With that in mind, it is important to gain deeper understanding about composition and development of resistance genes occurring in newborns to improve antibiotic stewardship measures. Current analysis displays lower detectability of resistance genes in rectal swabs than in meconium. It may be important to consider that microbial biomass in the newborn gut is low in comparison to later stages in life. Special caution needs to be set by the fact that bacterial mass within meconial samples varies greatly and may impact or even disable analysis of microbial composition (43). Thus, results need to be interpreted with caution regarding negative testing for resistance genes especially in rectal swabs, where the presence of skin microbes may simply displace DNA material present from actual gut microbes. Thus, the already-low total abundance of typical carriers of antimicrobial resistance genes (e.g., Enterobacter or Escherichia species) may be leading to resistance genes being below the detection limit in our assay in rectal swabs.

Of interest in our study is the presence of the *bla*_SHV_ gene, known to be a key carrier of ampicillin resistance (44), as this antibiotic is the most commonly used one in neonatal treatment (45). *bla*_SHV_ is known to be found in members of the family *Enterobacteriaceae*, especially in Klebsiella pneumoniae and Escherichia coli (46). Interestingly, both unclassified *Enterobacteriaceae* and E. coli were significantly higher abundant in meconium samples. We confirmed E. coli as a carrier of the *bla*_SHV_ gene in the respective sample by culture which corresponds to *bla*_SHV_ gene detection in the meconium microbiome in our data set. Thus, our results suggest that data obtained by analyzing abundance of resistance genes using rectal swabs provide results which greatly differ from the ones collected by meconium sample screening. This is underlining that the two sampling methods cannot be used interchangeably on the first days of life and rectal swabs may fail to identify critical resistance mechanisms present in the microbiome, knowledge which may prove valuable in preventing the spread of antibiotic resistance within the developing microbiome of a newborn (47).

In summary, our current study shows that rectal swab collection is not a reliable proxy compared to stool sampling on a newborn’s first days of life. Significant differences in alpha and beta diversity and variable taxonomic composition as well as the antibiotic resistance gene abundance profile indicate that these two sampling methods may present distinct gut microbiome and resistome compositions early after birth. We thus advise usage of meconium samples instead of rectal swabs in newborns, while evidence from previous research suggests rectal swabs are a sufficiently precise method for gut microbiome studies for adults.

## MATERIALS AND METHODS

### Study population and sample collection.

All samples were obtained during an exploratory randomized controlled trial at the University Hospital of Schleswig-Holstein (Campus Lübeck) carried out from January 2019 until June 2020 aiming to evaluate the impact of antimicrobial prophylaxis timing on the gut microbiome development in term-born infants (9), registration number DRKS00025305 at the German Registry of Clinical Studies (DRKS). All parents signed written informed consent. Ethical approval was granted by the ethics committee for research in human subjects of the University of Lübeck on 9 October 2018, reference number 18-264. In this study, we used a subset of 13 study participants for whom we had both first meconium passage samples and rectal swabs from the 1st day of life available following sequencing.

Rectal swabs were sampled by trained physicians or midwives using sterile dry Copan FLOQSwabs for drawing clinical samples (Copan Italia, Italy) on the 1st day of life. The tip of the swab was placed in an Eppendorf tube and frozen at −80°C. Meconium samples were collected as soon as meconium was passed and similarly frozen at −80°C until further laboratory processing.

### DNA isolation and sequencing.

Samples were thawed, and 500 μL of phosphate-buffered saline (PBS) was added to each tube with rectal swabs and vortexed for 5 min prior to further processing. Two hundred milligrams of meconium samples was taken after brief vortexing. All samples were processed using the DNeasy PowerSoil DNA isolation kit (Qiagen GmbH, Hilden, Germany). For each round of isolation, a negative control was included to control for contamination. Isolated DNA was stored at −20°C until further processing. To amplify partial sequences of the 16S rRNA gene, PCR with primers targeting V3/V4 hypervariable regions was performed as described elsewhere (9). Equimolar amounts of amplified samples were pooled and purified with the MinElute gel extraction kit (Qiagen GmbH, Hilden, Germany). The purified library was sequenced with the MiSeq platform (Illumina, San Diego, CA, USA) and MiSeq reagent kit V3 for 600 cycles. PhiX library and DNA isolation controls were included as positive and negative controls, respectively.

### Bioinformatic processing and statistical analysis.

Reads were processed using mothur (48), version 1.44.1, via the following pipeline: sequences with homopolymers under 12 and sizes shorter than 500 bp were aligned against the SILVA reference database (49), and nonaligned sequences were removed from further analysis. Chimeric sequences (VSEARCH [50]) were removed, and remaining sequences were taxonomically assigned using the Ribosomal Data Base (51) and rarefied at 1,800 reads/sample. Statistical analysis and graphical visualization were assembled via R (version 4.0.1) using the packages vegan (52), labdsv (53), BoutrosLab.plotting.general (54), OTUtable (55), RColorBrewer (56), and psych (57). Alpha diversity measurements were assessed using Shannon’s diversity index and the evenness index and calculating the number of detected species in each of the sample types. Differences between groups as well as differences in relative abundance were computed using a pairwise Wilcoxon rank sum test. False-discovery rate adjustment of *P* values was applied (58). In order to depict species with significantly different abundance between two sampling methods (*P* < 0.05), a heatmap visualizing Z-scores of average taxonomic abundances was produced. Correlation between alpha diversity parameters and interindividual variation was computed via Pearson’s correlation matrix from which *P* and *R*^2^ values were derived. Beta diversity was analyzed using principal-coordinate analysis generated with Bray-Curtis dissimilarities; to compare the impact of sampling method and interindividual variability, constrained correspondence analysis was used. Differences between groups were calculated via permutational multivariate analysis of variance using distance matrices.

### Detection of antibiotic resistance genes in rectal swabs and meconium samples.

To detect the abundance of various previously explored (9) resistance genes in both sample types, the subset of 13 paired meconium samples and rectal swabs was screened using PCR with specific primers (see Table S1 in the supplemental material) (59–62). Amplification was performed as described before (9). Depiction of resistance gene detection in both sample types was performed via a heatmap based on Euclidean distance estimation clustering. To confirm the presence of single bacterial isolates carrying the *bla*_SHV_ resistance gene, 100 mg of meconium sample was resuspended in 500 μL of PBS. Thereafter, 100 μL of 1:100 and 1:1,000 dilutions of the suspension was plated on 3 culturing media (Columbia agar plus 5% sheep blood, chocolate agar PolyViteX, and MacConkey agar) (bioMérieux, Marcy-l’Étoile, France) and cultured for 24 h at 37°C under aerobic and anaerobic conditions. Morphologically different colonies were isolated as pure cultures and identified with a matrix-assisted laser desorption ionization–time of flight (MALDI-TOF) mass spectrometer (Bruker Corporation, Billerica, MA, USA). Subsequently, for each of the detected species DNA isolation from 10 to 12 colonies was performed using a QIAamp DNA minikit (Qiagen GmbH, Hilden, Germany). Confirmation of the *bla*_SHV_ gene presence in the single bacterial isolates was carried out as described above for the meconium samples.

### Data availability.

The data that support the findings of this study are openly available in the European Nucleotide Archive at https://www.ebi.ac.uk/ena/browser/home, under accession number PRJEB47587.
