# Supplementary material for: First-Day-of-Life Rectal Swabs Fail To Represent Meconial Microbiota Composition and Underestimate the Presence of Antibiotic Resistance Genes
Source: Microbiol Spectr. 2023 Apr 25;11(3):e05254-22. doi: 10.1128/spectrum.05254-22 (PMC10269712; doi:10.1128/spectrum.05254-22)
Supplement: Supplemental file 1 — Fig. S1 and S2 and Table 1. Download spectrum.05254-22-s0001.docx, DOCX file, 1.8 MB [file spectrum.05254-22-s0001.docx]

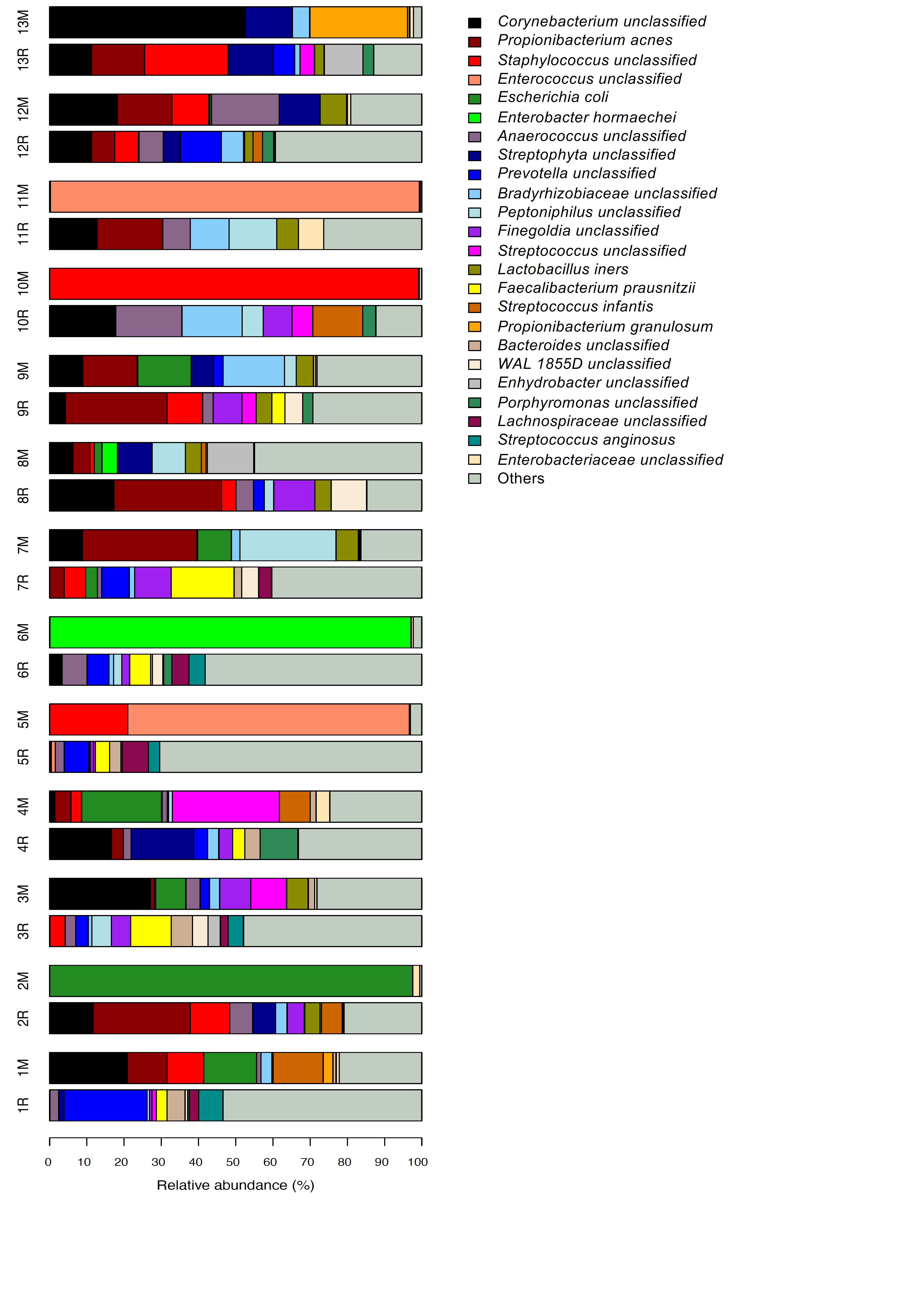


**Supplementary Figure 1: Individual pairwise comparison of relative abundance of rectal swabs and meconium samples.** Relative abundance of the 24 most prevalent taxa on genus level is given in both sampling types. Taxa with higher abundance are listed on the top of the legend. Y-axis gives sample assignment (participants by numbers, sample type by letters: R=rectal swab, M=meconium).


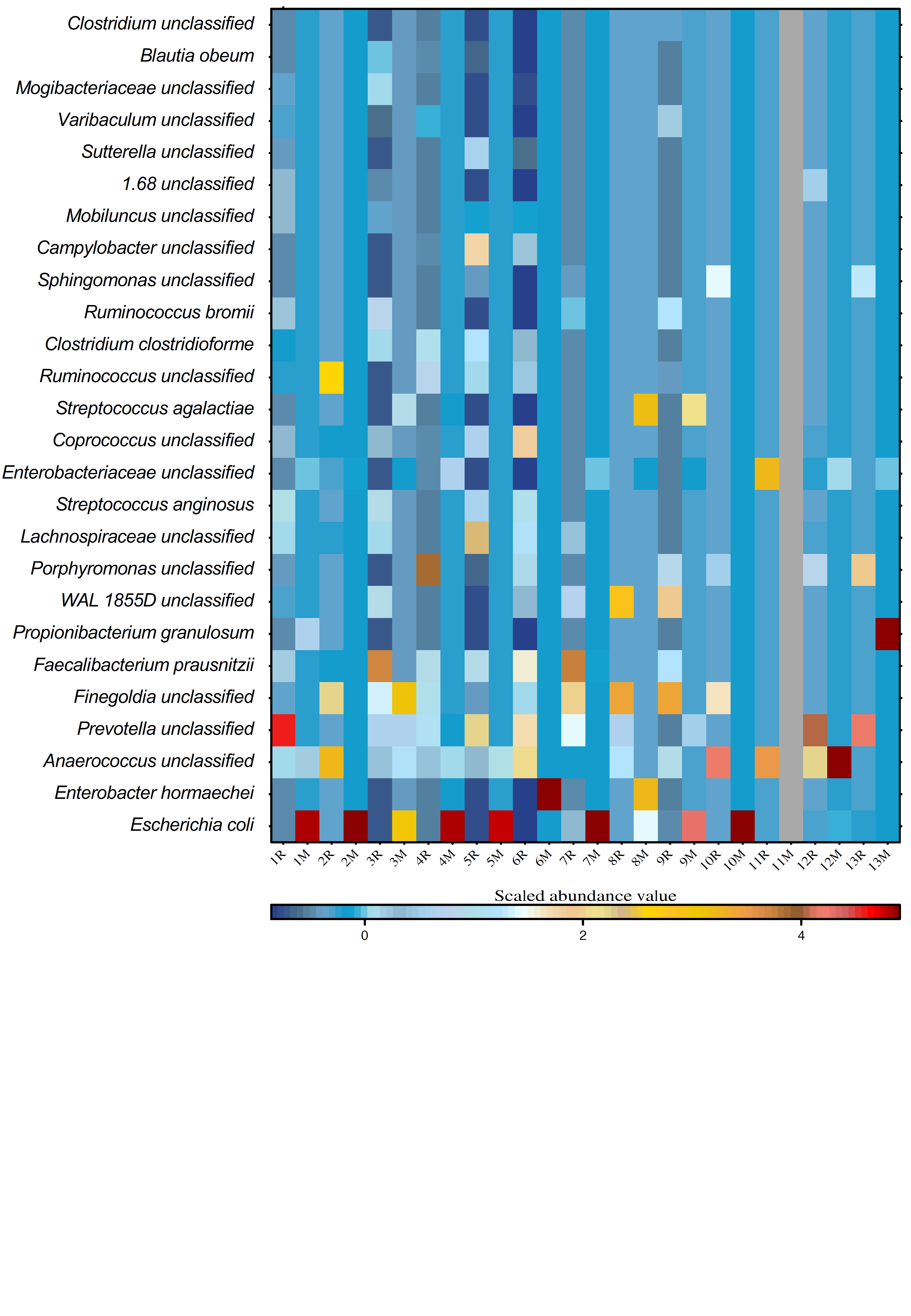


**Supplementary Figure 2: Heatmap displaying scaled abundance values of taxa being significant different between the two sampling methods based on individual samples.** Significance was calculated via pairwise Wilcoxon rank-sum test (p < 0.05, with use of false discovery rate adjustment of p-values). X-axis gives sample assignment (participants by numbers, sample type by letters: R=rectal swab, M=meconium).

**Supplementary table 1. Sequences of primer pairs used for resistance genes abundance screening**

| **Gene** | **Oligonucleotide** | | **Annealing, T** | **Awaited size, bp** | **Antibiotic group** | **Ref.** |
| --- | --- | --- | --- | --- | --- | --- |
|  | **Forward** | **Reverse** |  |  |  |  |
| *tet(W)* | AAGCGGCAGTCACTTCCTTCC | TCAAGTATCCCAGCGAAACC | 60 | 1239 | Tetracyclines | ^59^ |
| *tet(M)* | ACAGAAAGCTTATTATATAAC | TGGCGTGTCTATGATGTTCAC | 55 | 171 | Tetracyclines | ^59^ |
| *tet(O)* | ACGGARAGTTTATTGTATACC | TGGCGTATCTATAATGTTGAC | 60 | 171 | Tetracyclines | ^59^ |
| *tetA(B)* | TTGGTTAGGGGCAAGTTTTG | GTAATGGGCCAATAACACCG | 55 | 659 | Tetracyclines | ^59^ |
| *blatem* | TTTCGTGTCGCCCTTATTCC | CCGGCTCCAGATTTATCAGC | 60 | 690 | Penicillins | ^59^ |
| *blaCTX-M* | ATGTGCAGYACCAGTAARGTKATGGC | GGGTRAARTARGTSACCAGAAYSAGCGG | 60 | 592 | Penicillins | ^59^ |
| *blaSHV* | CACTCAAGGATGTATTGTG | TTAGCGTTGCCAGTGCTCG | 58 | 885 | Penicillins | ^59^ |
| *mecA* | GGGATCATAGCGTCATTATTC | AACGATTGTGACACGATAGCC | 56 | 527 | Penicillins | ^59^ |
| *aac(6")-le-aph(2")* | CCAAGAGCAATAAGGGCATACC | CACACTATCATAACCATCACCG | 55 | 222 | Aminoglycosides | ^59^ |
| *strA* | CTTGGTGATAACGGCAATTC | CCAATCGCAGATAGAAGGC | 65 | 548 | Aminoglycosides | ^59^ |
| *cmlA1* | CACCAATCATGACCAAG | GGCATCACTCGGCATGGACATG | 60 | 115 | Chloramphenicol | ^59^ |
| *blaCMY* | GATTCCTTGGACTCTTCAG | TAAAACCAGGTTCCCAGATAGC | 55 | 1807 | Penicillins | ^60^ |
| *qnr(A)* | ATTTCTCACGCCAGGATTTG | GATCGGCAAAGGTTAGGTCA | 55 | 516 | Quinolones | ^61^ |
| *vanA* | GGGAAAACGACAATTGC | GTACAATGCGGCCGTTA | 55 | 732 | Vancomycin | ^62^ |
| *vanB* | ACCTACCCTGTCTTTGTGAA | AATGTCTGCTGGAACGATA | 55 | 300 | Vancomycin | ^62^ |
